# Low concordance of oral and genital HPV infection among male patients with sexually transmitted infections in Vietnam

**DOI:** 10.1186/s12879-019-4175-0

**Published:** 2019-07-04

**Authors:** Hai Ha Long Le, Xiuqiong Bi, Azumi Ishizaki, Hung Van Le, Trung Vu Nguyen, Hiroshi Ichimura

**Affiliations:** 1 Department of Viral Infection and International Health, Graduate School of Medical Sciences, Kanazawa University, 13-1 Takara-machi, Kanazawa, 920-8640 Japan; 2 Graduate School of Advanced Preventive Medical Sciences, Kanazawa University, Kanazawa, Japan; 3 Hanoi Medical University, Hanoi, Vietnam; 4 National Hospital of Dermatology and Venereology, Hanoi, Vietnam

**Keywords:** HPV, STI, Vietnam, Concordance

## Abstract

**Background:**

Human papillomavirus (HPV) causes cancers in men, including penile, anal, and oropharyngeal cancers. This cross-sectional study aimed to investigate the prevalence, the genotypes, and the risk factors of HPV infections in the oral cavity, compared to those in the genitals, among males diagnosed with sexually transmitted infections (STIs) in Vietnam.

**Methods:**

Oral, urinary, penile, and urethral samples were collected from 198 male Vietnamese patients with STIs (median age 31.0 years, range 17–68). HPV DNA was isolated and amplified with PCR, with modified and/or original GP5^+^/GP6^+^ primers. Samples were genotyped with a gene array assay and/or population sequencing.

**Results:**

HPV DNA was detected in 69 (34.8%) of 198 patients. Of these, 16 patients (8.1%) had infections in the oral cavity and 58 (29.3%) had infections in the genitals (4.5% in the urine, 25.8% in the penis, and 8.1% in the urethra). The concordance of HPV infections between the oral cavity and the genitals was poor (kappa = 0.01). Of the 16 patients with oral HPV DNA, 11 (68.8%) had no HPV DNA in the genitals. In the remaining five patients, HPV DNA was found at both sites, but only one showed similar strains at both sites. In the other four patients, the HPV genotypes were completely discordant between these sites. HPV18 was the most common high-risk HPV genotype in both oral (9/16, 56.3%) and genital (10/58, 17.2%) sites. Multivariable analyses showed that older age (OR 1.05), higher education (OR 2.17), and no knowledge of STIs (OR 4.21) were independent risk factors for genital HPV infections; in contrast, only older age (OR 1.05) was an independent risk factor for oral HPV infections.

**Conclusions:**

The low concordance of HPV genotypes between oral and genital infection sites suggested that the acquisition, persistence, and/or clearance of HPV infections were different between these sites. Although HPV DNA was detected significantly less frequently in oral samples than in genital samples, oral samples should also be used for HPV screening in men.

**Electronic supplementary material:**

The online version of this article (10.1186/s12879-019-4175-0) contains supplementary material, which is available to authorized users.

## Background

Human papillomavirus (HPV) causes the most common sexually transmitted infection (STI) worldwide. An estimated 660 million people are infected globally, including about 370 million men [1, 2]. In men, HPV causes cancers, including penile, anal, and oropharyngeal cancers [3, 4]. In women, HPV causes cervical cancer, the fourth most frequent cancer among women worldwide [5]. Globally, in 2012, there were an estimated 66,000 new cases of HPV-related cancers in men [4], including 17,000 anal cancers, 13,000 penile cancers, and 24,000 oropharyngeal cancers [4, 6]. It is noteworthy that the number of new HPV-related oropharyngeal cancer cases in men increased from 17,000 in 2008 to 24,000 in 2012, worldwide [6, 7].

To prevent HPV infections, and the subsequent development of cancer in men, it is important to know the status of HPV infections in the oral cavity and in the genitals. Previous studies have reported that the prevalences of concurrent HPV infections in the oral cavity and the genitals were 1.9 and 8.8%, among general male population in China and the United States, respectively [8, 9]; 9% in heterosexual men with human immunodeficiency virus (HIV) infections in Spain [10]; and 0–7% in men who have sex with men (MSM), with and without HIV infections, in the United Kingdom, Spain, and Greece [10–12]. Among the general Chinese and American male persons concurrently infected with HPV in oral and genital sites, 36.2–60.5% showed similar or identical HPV genotype(s) in the two sites [8, 9, 13]. The frequencies of sexual contact and autoinoculation were reportedly related to the frequencies of oral HPV infections in young men in the United States [13]. However, the profile of oral HPV infections currently remains unclear. Specifically, it remains unclear whether oral and genital HPV infections occur simultaneously or whether HPV autoinoculation from the genitals to the oral cavity, and vice versa, can occur in men.

In Vietnam, there is limited information on genital HPV infections among men. It was reported that the prevalence of HPV was 25.0% among male patients with STIs; moreover, HPV52 (18.8%) was the most prevalent high-risk genotype, followed by HPV18 (16.7%) and HPV16 (6.3%) [14]. The prevalence of HPV was 23.0–79.6% among patients with penile cancer, and HPV16 (67.4–89.0%) was the most prevalent high-risk genotype [15, 16]. Regarding oral HPV infections, only one study showed that, among women at risk of STIs, the oral HPV prevalence was 24.6% [17]. No study has reported the status of oral HPV infections or the relationship between oral and genital HPV infections among Vietnamese men.

The present study aimed to investigate the prevalence and profile of HPV infections in the oral cavity and compared them to the HPV infections in the genitalia of male patients with STIs, in Hanoi, Vietnam. We also analyzed some potential risk factors for oral and genital HPV infections.

## Methods

### Subjects

From June to August 2015 and from December 2015 to February 2016, we recruited all male patients with an STI diagnosis that visited the National Hospital of Dermatology and Venereology, Hanoi, Vietnam. Of 1532 men diagnosed with STIs, 220 (14.4%) provided written informed consent to participate in this study. Patients were interviewed with a questionnaire to collect data on potential risk factors for HPV infections. The inclusion criteria were male patients with any STI or STI-related symptoms, based on the clinical examination request forms at the hospital. We excluded patients with condyloma (*n* = 10) to avoid a bias in the HPV genotype distribution, because more than 90% of condylomas are caused by HPV6 and HPV11. Thus, a total of 210 male patients with STIs were included in this study. The study protocol was reviewed and approved by the Ethics Committees of the National Hospital of Dermatology and Venereology, Hanoi, Vietnam (No. 113335143) and Kanazawa University, Japan (No. 1612–2).

### Sample collection

Penile, urethral, and urinary samples were collected from the 210 patients, as described previously [14]. Briefly, penile and urethral swabs were obtained with nylon tips soaked in saline solution (FLOQSwab R100 and FLOQSwab U80, respectively; Copan Diagnostics Inc., Murrieta, CA). The swabs were soaked in 1.5 ml LiquiPrep preservation solution (LGM International Inc., Fort Lauderdale, FL), and the released-cell suspension was stored at 4 °C until use. We also collected 30 ml of midstream urine, to avoid contamination by urethral cells. Oral samples were collected by asking patients to gargle with 15 ml of phosphate buffered saline with the face pointing upward for about 10 s, then spitting the solution into a receptacle. Urinary and oral samples were centrifuged at 1500 *g* for 10 min, and the cell pellets were resuspended in 3 ml and 2.5 ml of LiquiPrep solution, respectively. These samples were stored at 4 °C until use [14, 18].

### DNA extraction

DNA was extracted from the cell suspensions as described previously [14]. Briefly, after the cell suspension was centrifuged, the cell pellet was washed twice with E-MEM (Wako Pure Chemical Industries, Ltd., Osaka, Japan) and resuspended in 50 μl of E-MEM. DNA was extracted from the cells with a DNA extraction kit (SMI Test; Genome Science Laboratories, Fukushima, Japan). The quality of the extracted DNA was confirmed, based on PCR amplification of the glyceraldehyde-3-phosphate dehydrogenase (GAPDH) gene [14].

### HPV screening

To detect HPV DNA, the qualified DNA samples were screened in a PCR assay with the modified GP5^+^/GP6^+^ primers, as described previously [14]. The presence of HPV DNA was confirmed with agarose gel electrophoresis and ethidium bromide staining. HPV DNA-negative samples were retested with the original GP5^+^/GP6^+^ primers, as described previously [14].

### HPV genotyping

HPV genotypes were determined with the 21 HPV GenoArray Diagnostic Kit (Hybribio, Chaozhou, China), which could genotype HPV16, 18, 31, 33, 35, 39, 45, 51–53, 56, 58, 59, 66, 68, 6, 11, 42–44, and 81, according to the manufacturer’s instructions. HPV DNA-positive samples that could not be genotyped with this kit were sequenced and genotyped with the BLAST program (U.S. National Library of Medicine, Bethesda, MD), as described previously [14]. The detected HPV genotypes were classified into high-risk (HPV16, 18, 31, 33, 35, 39, 45, 51, 52, 56, 58, and 59), probably high-risk (HPV68), possibly high-risk (HPV30, 53, and 66), or low-risk (HPV6, 11, 40, 42, 43, 44, 74, 76, 81, 90, and 114) [19] groups. For this study, high-risk and probably high-risk HPV types were classified as “high-risk”, and possibly high-risk and low-risk HPV types were classified as “low-risk” in further analyses.

### Statistical analysis

Statistical analyses were performed with SPSS Version 22 for Windows. Cohen’s kappa value was calculated to check the concordance of HPV infections between paired samples from the same patients. McNemar’s test was used to assess differences between paired samples in the rates of detecting HPV DNA and the specific HPV genotypes [8]. We assessed the association between oral and genital HPV infections with a binary logistic regression analysis. To investigate associations between potential risk factors and HPV infections, we performed a multivariable analysis with a stepwise binary logistic regression model and a *P*-value cut-off point of 0.20. Some factors such as “age”, “age at sexual debut”, “lifetime number of sexual partners” and “number of sexual partners in the last 6 months” were analyzed as continuous variables. *P* values < 0.05 were considered statistically significant.

The classification of “genital HPV infection” was defined as the positive detection of at least one HPV genotype in the penile, urethral, and/or urinary sample. The classification of “overall HPV infection” was defined as the positive detection of at least one HPV genotype in the oral and/or genital samples.

## Results

### Subjects used for the analyses

GAPDH DNA was successfully amplified in 96.7% (203/210) of the penile samples, 100% (210/210) of the urethral samples, 99.5% (209/210) of the urinary samples, and 97.6% (205/210) of the oral samples. Overall, 198 male patients with STIs (median age 31.0 years, range 17–68) had positive GAPDH DNA results in all four samples. These samples and data were used in further analyses. Of the 198 patients, 118 had urethral discharge, 42 had urethritis, five had gonorrhea, and six had dysuria; these patients comprised the urethritis group (*n* = 171). The other patients included 20 with balanitis, three with molluscum contagiosum, two with genital ulcer, one with tuberculosis of testis, and one with foreskin itchiness; these comprised the non-urethritis group (*n* = 27).

### HPV infection profile

Of the 198 patients included, 69 (34.8%) displayed HPV DNA in at least one of the four (penile, urethral, urinary and oral) samples. The prevalence of HPV DNA was 8.1% (16 cases) in the oral cavity and 29.3% (58 cases) in the genitals (including 25.8% in the penis, 8.1% in the urethra, and 4.5% in the urine). The HPV prevalence was significantly lower in the oral cavity than in the genitals (*P* < 0.01). In the genitals, the HPV prevalence was significantly higher in the penis than in the urethra and urine (both *P* < 0.01; Table 1).

**Table 1:** Comparison of HPV detection between genital and oral samples and among genital samples (*n* = 198)

	No. of samples with								
Samples compared	Indicated paired results^a^				% Agreement			Cohen’s Kappa	McNemar
	+/+	–/–	+/–	–/+	Overall	Positive	Negative	(95% CI)	*P* value
Between genital and oral									
Genital/oral	5	129	53	11	67.7	13.5	80.1	0.01 (−0.09–0.12)	< 0.01
Penile/oral	5	136	46	11	71.2	14.9	82.7	0.03 (−0.07–0.16)	< 0.01
Urethral/oral	1	167	15	15	84.8	6.3	91.8	−0.02 (−0.11–0.13)	1.00
Urinary/oral	1	174	8	15	88.4	8.0	93.8	0.02 (−0.08–0.19)	0.21
Among genitals									
Penile/urethral	10	141	41	6	76.3	29.9	85.7	0.20 (0.06–0.35)	< 0.01
Penile/urinary	8	146	43	1	77.8	26.7	86.9	0.21 (0.09–0.34)	< 0.01
Urethral/urinary	8	181	8	1	95.5	64.0	97.6	0.62 (0.34–0.83)	0.04

Cohen’s Kappa: assessment of the concordance of HPV infection between paired samples

McNemar test: assessment of the difference in the prevalence of HPV infection between paired samples

+, presence of any HPV genotype; –, absence of all HPV genotypes; CI: confidence interval

^a^The result indicated on either side of each slash corresponds to the type listed in the same position in the first column

Among the samples from the 69 patients with HPV infections, we detected 121 HPV strains (102 in genital and 19 in oral samples) and 27 different HPV genotypes (13 high-risk and 14 low-risk; Table 2, Additional file 1: Table S1). Of these 69 patients, 50 (72.5%) were infected with high-risk, and 41 (59.4%) with low-risk HPVs. The most common high-risk genotype was HPV18 (27.5%), followed by HPV52 (14.5%), HPV16 (10.1%), and HPV68 (10.1%; Table 2, Additional file 1: Table S1).

**Table 2:** Proportion of HPV genotypes in HPV positive patients

HPV type	Genitals, *n* = 58 (%) [95% CI]	Oral cavity, *n* = 16 (%) [95% CI]	Overall, *n* = 69 (%) [95% CI]
High-risk^a^	42 (72.4) [58.9–83.0]	12 (75.0) [47.4–91.7]	50 (72.5) [60.2–82.2]
16	6 (10.3) [4.3–21.8]	1 (6.3) [0.3–32.3]	7 (10.1) [4.5–20.4]
18	10 (17.2) [9.0–29.9]	9 (56.3) [30.6–79.3]	19 (27.5) [17.8–39.8]
31	2 (3.4) [0.6–13.0]	0	2 (2.9) [0.5–11.0]
33	1 (1.7) [0.1–10.5]	0	1 (1.4) [0.1–8.9]
35	1 (1.7) [0.1–10.5]	0	1 (1.4) [0.1–8.9]
39	4 (6.9) [2.2–17.6]	1 (6.3) [0.3–32.3]	5 (7.2) [2.7–16.8]
45	1 (1.7) [0.1–10.5]	0	1 (1.4) [0.1–8.9]
51	5 (8.6) [3.2–19.7]	0	5 (7.2) [2.7–16.8]
52	10 (17.2) [9.0–29.9]	0	10 (14.5) [7.5–25.5]
56	2 (3.4) [0.6–13.0]	1 (6.3) [0.3–32.3]	3 (4.3) [1.1–13.0]
58	5 (8.6) [3.2–19.7]	0	5 (7.2) [2.7–16.8]
59	4 (6.9) [2.2–17.6]	1 (6.3) [0.3–32.3]	5 (7.2) [2.7–16.8]
68	7 (12.1) [5.4–23.9]	0	7 (10.1) [4.5–20.4]
Low-risk^a^	36 (62.1) [48.4–74.2]	6 (37.5) [16.3–64.1]	41 (59.4) [46.9–70.9]
30	1 (1.7) [0.1–10.5]	0	1 (1.4) [0.1–8.9]
53	3 (5.2) [1.3–15.3]	1 (6.3) [0.3–32.3]	4 (5.8) [1.9–14.9]
66	1 (1.7) [0.1–10.5]	1 (6.3) [0.3–32.3]	2 (2.9) [0.5–11.0]
6	7 (12.1) [5.4–23.9]	0	7 (10.1) [4.5–20.4]
11	7 (12.1) [5.4–23.9]	2 (12.5) [2.2–39.6]	8 (11.6) [5.5–22.1]
40	1 (1.7) [0.1–10.5]	0	1 (1.4) [0.1–8.9]
42	2 (3.4) [0.6–13.0]	1 (6.3) [0.3–32.3]	3 (4.3) [1.1–13.0]
43	5 (8.6) [3.2–19.7]	0	5 (7.2) [2.7–16.8]
44	1 (1.7) [0.1–10.5]	0	1 (1.4) [0.1–8.9]
74	2 (3.4) [0.6–13.0]	0	2 (2.9) [0.5–11.0]
76	1 (1.7) [0.1–10.5]	0	1 (1.4) [0.1–8.9]
81	11 (19.0) [10.3–31.8]	1 (6.3) [0.3–32.3]	12 (17.4) [9.7–28.8]
90	2 (3.4) [0.6–13.0]	0	2 (2.9) [0.5–11.0]
114	1 (1.7) [0.1–10.5]	0	1 (1.4) [0.1–8.9]
Total strains	103	19	121

*CI* confidence interval

^a^“High-risk” and “low-risk” HPV infection were defined as a positive result for at least one of high-risk and low-risk HPV genotypes, respectively

Of the 16 patients with oral HPV infections, 12 (75.0%) were infected with high-risk and six (37.5%) with low-risk HPVs. HPV18 was most common in the oral cavity (56.3%, 9/16). Of the 58 patients with genital HPV infections, 42 (72.4%) were infected with high-risk and 36 (62.1%) with low-risk HPVs. HPV18 and HPV52 were the most common high-risk HPVs (both 17.2%, 10/58) in the genitals (Table 2). The prevalence of HPV18 was not significantly different between the oral cavity (4.5%) and the genitals (5.1%, *P* = 0.50).

The concordance of HPV infections between the oral cavity and the genitals was poor (kappa = 0.01, Table 1). Of the 16 patients with oral HPV infections, 11 (68.8%) were negative for HPV DNA in the genitals. Of the five patients that had HPV DNA at both sites, four had completely discordant HPV genotypes between the two sites. The remaining patient had three HPV genotypes (HPV6, 11, and 81) in the genitals and two (HPV11 and 18) in the oral cavity; thus, only HPV11 was concordant between the two sites (Additional file 1: Table S1). Therefore, among 198 male patients with STIs, only one (0.5%) had concordant HPV genotypes in oral and genital sites. Among the genital sites, HPV infections were slightly concordant between the penis and the urethra (kappa = 0.20) and between the penis and the urine (kappa = 0.21), but the concordance was good between the urethra and the urine (kappa = 0.62) (Table 1).

Oral HPV infections were found in 8.6% (5/58) of patients with genital HPV infections and in 7.9% (11/140) of patients without genital HPV infections (Additional file 1: Table S1). This difference was not significant (odds ratio [OR] 1.11, 95% confidence interval [CI]: 0.37–3.34, *P* = 0.86).

### Risk factors for HPV infections

Potential risk factors for HPV infections in the oral cavity and the genitals were analyzed based on responses to the questionnaire. The multivariable analysis showed that, overall, older age (OR 1.04, 95% CI: 1.02–1.07, *P* < 0.01) and higher education (OR 2.02, 95% CI: 1.06–3.84, *P* = 0.03) were associated with HPV infections in male patients with STIs. HPV infections were not associated with other factors, such as occupation, smoking, alcohol consumption, a family member with cancer, circumcision, age at sexual debut, total number of sexual partners, number of sexual partners in the last 6 months, a female sex worker as a sexual partner in the last 6 months, condom usage, STI history, or the type of STI (urethritis or non-urethritis; Additional file 1: Table S2). Genital HPV infections were significantly associated with older age (OR 1.05, 95% CI: 1.01–1.08, *P <* 0.01), higher education (OR 2.17, 95% CI: 1.08–4.35, *P* = 0.03), and lack of knowledge about STIs (OR 4.21, 95% CI: 1.08–16.42, *P* = 0.04; Table 3). In contrast, oral HPV infections were significantly associated only with older age (OR 1.05, 95% CI: 1.01–1.10, *P* = 0.02) (Table 4).

**Table 3:** Risk factors for genital HPV infection

Factors	Infections/cases	Unadjusted OR (95%CI)	*P*	Adjusted OR (95% CI)	*P*
Age					
Older age	58/198	1.03 (1.01–1.06)	**0.02**	1.05 (1.01–1.08)	**0.01**
Marital status					
No	23/75	1		1	
Yes	35/123	0.90 (0.48–1.69)	0.74	0.55 (0.25–1.21)	0.14
Occupation					
Unstable or no job	16/53	1			
Blue collar	11/47	0.71 (0.29–1.73)	0.45	–	–
White collar	31/98	1.07 (0.52–2.21)	0.86	–	–
Education					
< College	17/84	1		1	
≥ College	41/114	2.21 (1.15–4.26)	**0.02**	2.17 (1.08–4.35)	**0.03**
Smoking					
No	39/123	1			
Yes	19/75	0.73 (0.38–1.39)	0.34	–	–
Alcohol consumption					
No	5/24	1			
Yes	53/174	1.66 (0.59–4.69)	0.34	–	–
Family member with cancer					
No	53/175	1			
Yes	5/23	0.64 (0.23–1.81)	0.40	–	–
STI knowledge					
Yes	5/10	1		1	
No	53/188	2.55 (0.71–9.16)	0.15	4.21 (1.08–16.42)	**0.04**
Circumcision					
No	41/139	1			
Yes	17/59	0.97 (0.50–1.90)	0.92	–	–
Age at sexual debut					
Older age	58/198	1.03 (0.93–1.13)	0.58	–	–
Total number of sexual partners					
Higher number	58/198	1.00 (0.99–1.02)	0.68	–	–
Number of sexual partners in last 6 months					
Higher number	58/198	0.98 (0.93–1.05)	0.60	–	–
Female sex worker as a sexual partner in last 6 months					
No	46/164	1			
Yes	12/34	1.40 (0.64–3.06)	0.40	–	–
Condom usage					
Rarely or Never	10/48	1			
Sometimes	34/104	1.85 (0.82–4.14)	0.14	–	–
Everytime	14/46	1.66 (0.65–4.25)	0.29	–	–
STI history					
No	17/66	1			
Yes	41/132	1.30 (0.67–2.52)	0.44	–	–
Urethritis					
No	8/27	1			
Yes	50/171	0.98 (0.40–2.39)	0.97	–	–
Oral sex					
No	38/126	1			
Yes	20/72	0.89 (0.47–1.69)	0.72	–	–

*STI* sexually transmitted infection, *OR* odds ratio, *CI* confidence interval, Dash (-) indicates not included in the last step of the stepwise binary logistic regression analysis

**Table 4:** Risk factors for oral HPV infection

Factors	Infections/cases	Unadjusted OR (95% CI)	*P*	Adjusted OR (95% CI)	*P*
Age					
Older age	16/198	1.05 (1.01–1.09)	**0.02**	1.05 (1.01–1.10)	**0.02**
Marital status					
No	2/75	1			
Yes	14/123	4.69 (1.04–21.24)	**0.05**	–	–
Occupation					
Unstable or no job	2/53	1			
Blue collar	4/47	2.37 (0.41–13.59)	0.33	–	–
White collar	10/98	2.90 (0.611–13.75)	0.18	–	–
Education					
< College	6/84	1			
≥ College	10/114	1.25 (0.44–3.59)	0.68	–	–
Smoking					
No	9/123	1			
Yes	7/75	1.30 (0.46–3.66)	0.61	–	–
Alcohol consumption					
No	1/24	1			
Yes	15/174	2.17 (0.27–17.21)	0.46	–	–
Family member with cancer					
No	12/175	1		1	
Yes	4/23	2.86 (0.84–9.76)	0.09	3.45 (0.97–12.29)	0.06
STI knowledge					
Yes	15/188	1			
No	1/10	1.28 (0.15–10.81)	0.82	–	–
Circumcision					
No	9/139	1			
Yes	7/59	1.94 (0.69–5.49)	0.21	–	–
Age at sexual debut					
Older age	16/198	1.11 (0.96–1.28)	0.18	–	–
Total number of sexual partners					
Higher number	16/198	1.00 (0.98–1.03)	0.83	–	–
Number of sexual partners in last 6 months					
Higher number	16/198	0.88 (0.64–1.23)	0.46	–	–
Female sex worker as a sexual partner in last 6 months					
No	16/164	1			
Yes	0/34	NE (NE-NE)	1.00	–	–
Condom usage					
Rarely or Never	3/48	1			
Sometimes	11/104	1.77 (0.47–6.68)	0.40	–	–
Every time	2/46	0.68 (0.11–4.28)	0.68	–	–
STI history					
No	4/66	1			
Yes	12/132	1.55 (0.48–5.01)	0.46	–	–
Urethritis					
No	1/27	1			
Yes	15/171	2.50 (0.32–19.74)	0.39	–	–
Oral sex					
No	10/126	1			
Yes	6/72	1.06 (0.37–3.03)	0.92	–	–

*STI* sexually transmitted infection, *OR* odds ratio, *CI* confidence interval, *NE* not estimable, Dash (-) indicates not included in the last step of the stepwise binary logistic regression analysis

## Discussion

In this study, we investigated the prevalence and genetic profiles of HPV infections in the oral cavity and compared them to the prevalence and genetic profiles of HPV infections in the genitals of male patients diagnosed with STIs in Hanoi, Vietnam. We found a significantly lower prevalence of HPV infections in the oral cavity than in the genitals (8.1% vs. 29.3%). Moreover, we observed low concordance between the types of HPV infections (0.5%) in these two anatomical sites in our cohort. HPV18 was the most common high-risk genotype in both sites. To our knowledge, this study was the first to compare the prevalence and concordance of HPV infections between oral and genital sites in Vietnamese men.

Among males with STIs in Hanoi, the prevalence of oral HPV infections was 8.1%, which was higher than that observed in Greece (3.7%) [12], but lower than that observed in the United States (15.3%) [20], Japan (18.8%) [18], and Italy (37.0%) [21]. Information about oral HPV infections among Vietnamese women was available from only a single study. That study showed that the oral HPV prevalence was 24.6% among women at risk for STIs in Ho Chi Minh City, Vietnam [17], which was higher than that of males with STIs in Hanoi, Vietnam (8.1%, *P* < 0.01). It was difficult to compare these studies directly, due to differences in the study subjects; age distributions; sample collection, processing, and storage; DNA extraction methods; and/or the sensitivity and specificity of HPV DNA detection methods. However, our observations suggested that HPV prevalence could vary according to geographical area, population ethnicity, and even the sex of individuals residing in the same country.

In the present study, HPV DNA was detected in the oral cavity significantly less frequently than in the genitals (8.1% vs. 29.3%, *P* < 0.01). This result is consistent with those of other studies among men that attended STI and HIV clinics in Greece (3.7% vs. 22.8%) [12] and among men in the general population in the United States (11.2% vs. 45.3%) [9]. The lower prevalence of oral compared to genital HPV infections might be explained by differences in the acquisition, persistence, and clearance of HPV between the two sites. For example, antimicrobial agents might be present in saliva [8]; HPV-infected cells might be eliminated during eating, drinking, and rinsing the mouth; and there might be different site-specific immune responses [9].

The current study also showed that the concordance of specific HPV genotypes was low between oral and genital sites (0.5%, kappa = 0.01). Of the five patients with HPV-positive samples in both sites, only one patient showed similar genotypes between sites, and no patient showed complete genotype concordance between the two sites. This finding was consistent with previous studies, which showed low or no concordance (0–3.2%) between oral and genital sites in an MSM cohort in the United Kingdom [11], in men that attended STI and HIV clinics in Greece [12], and in healthy men in the United States [9] and China [8]. The low concordance of HPV genotypes between oral and genital sites suggested that HPV infections are rarely transferred from the genitals to the oral cavity, and vice versa. A longitudinal study would be necessary to clarify the differences in acquisition, persistence, and/or clearance of HPV infections at these sites.

In the current study, the prevalence of oral HPV infections did not differ between patients with and without genital HPV infections (8.6% vs. 7.9%, *P* = 0.86). This result was consistent with a previous study in the United Kingdom [11], which showed that the oral HPV prevalence did not differ between MSM individuals with and without anogenital HPV infections (14.3% vs. 13.2%). In contrast, it was reported that the prevalence of oral HPV infections was higher among healthy men with genital infections than among those without genital HPV infections in rural China (11.4% vs. 5.7%) [8] and in the United States (19.3% vs. 4.4%) [9]. These findings suggested that genital HPV infections may not be a risk factor for oral HPV infections in men at high risk, such as male patients with STIs and MSM individuals, and that autoinoculation of HPV from the genitals to the oral cavity did not typically occur, even in men at high risk.

The multivariable analysis showed that older age and higher education were independent risk factors for genital HPV infections, but that only older age was an independent risk factor for oral HPV infections in male patients with STIs in Vietnam. These findings could be explained by the association between older age and a weakened immune system [9], the reactivation of latent HPV infections [9], and a greater total number of sexual partners. In addition, people with a higher education are likely to have relatively higher incomes and are likely to have more sexual partners, which increases the likelihood of becoming infected with HPV.

This study had several limitations. First, the sample size was limited. In this study, we analyzed the baseline data of an ongoing longitudinal study. It was difficult to recruit patients in the longitudinal study, because most did not want to return regularly to the hospital after the disease was cured, and some could not come back, because they resided in distant areas. Second, there was no gold standard method for collecting oral HPV specimens. Third, it was unclear whether the sensitivity of HPV DNA testing was similar at different sites and in different sample types. However, we only analyzed DNA samples that were qualified with PCR by targeting the GAPDH gene. Fourth, some risk factors, such as oral sex experience and the number of sexual partners, might be underreported, due to recall error and social stigma. These limitations could have affected our ability to detect some associations.

## Conclusions

This study was the first to demonstrate the prevalence of oral HPV infections among Vietnamese men and investigate the association between genital and oral HPV infections. The low concordance between HPV infections at these sites suggested that there might be differences in the acquisition, persistence, and/or clearance of HPV infections at these sites. Although HPV DNA was detected significantly less frequently in oral samples than in genital samples, oral samples should also be used for HPV screening in men.

## Additional file

Additional file 1: **Table S1.** HPV genotypes in different samples in each HPV DNA positive patients. **Table S2**. Risk factors for overall HPV infection. (DOCX 25 kb)

## Data Availability

The data sets used and/or analyzed during the current study are available from the corresponding author at a reasonable request.

## Abbreviation

CI: Confidence interval
GAPDH: Glyceraldehyde-3-phosphate dehydrogenase
HPV: Human papillomavirus
MSM: Men who have sex with men
NE: Not estimable
OR: Odds ratio
STI: Sexually transmitted infections
