# Supplementary material for: Low concordance of oral and genital HPV infection among male patients with sexually transmitted infections in Vietnam
Source: BMC Infect Dis. 2019 Jul 4;19:578. doi: 10.1186/s12879-019-4175-0 (PMC6610792; doi:10.1186/s12879-019-4175-0)
Supplement: Supplementary file 1 — Table S1. HPV genotypes in different samples in each HPV DNA positive patients. Table S2. Risk factors for overall HPV infection. (DOCX 25 kb) [file 12879_2019_4175_MOESM1_ESM.docx]

**Additional file 1: Table S1.** HPV genotypes in different samples in each HPV DNA-positive patient

| **Patient Number** | **HPV genotypes** | | | |
| --- | --- | --- | --- | --- |
|  | **Urinary (n=9)** | **Penile (n=51)** | **Urethral (n=16)** | **Oral (n=16)** |
| 1 | **58**, **68** | **35**, 53, **56**, **58**, **68** | **35**, **58**, **68** | **18** |
| 2 | **58** | 11, **33**, **58**, **68** | 11, **58** | - |
| 3 | 6, **58** | 6, 11, **18**, **52**, **58**, **68**, 81 | 6 | - |
| 4 | 42 | 43, **51** | 43 | - |
| 5 | - | 44, **51**, **52**, 53 | - | **18** |
| 6 | - | **16**, **58** | **56** | - |
| 7 | - | **18** | **18**, 42 | - |
| 8 | - | 11, **59** | - | **18** |
| 9 | - | 6, 11, 81 | - | 11, **18** |
| 10 | - | 6, **52**, 66, **68** | - | - |
| 11 | - | 6, 43 | - | - |
| 12 | - | 6, **18**, **52** | - | - |
| 13 | - | 6, **16**, **39**, 81 | - | - |
| 14 | - | 6, **16** | - | - |
| 15 | - | 43, **51** | - | - |
| 16 | - | **39**, 81 | - | - |
| 17 | - | **18**, 81 | - | - |
| 18 | - | **18**, 81 | - | - |
| 19 | - | **18**, 81 | - | - |
| 20 | - | **18**, **52**, **68** | - | - |
| 21 | - | 11, **59** | - | - |
| 22 | - | 11, **16** | - | - |
| 23 | - | - | - | **16**, **18** |
| 24 | - | - | - | **59**, 81 |
| 25 | **31** | 40 | **31** | - |
| 26 | 74 | 74 | **45** | - |
| 27 | **16** | **16** | **16** | - |
| 28 | **58** | **58** | **58** | - |
| 29 | 81 | - | - | - |
| 30 | - | **31** | - | **39** |
| 31 | - | 74 | - | - |
| 32 | - | **59** | - | - |
| 33 | - | **39** | - | - |
| 34 | - | 11 | - | - |
| 35 | - | **18** | - | - |
| 36 | - | 76 | - | - |
| 37 | - | 81 | - | - |
| 38 | - | **52** | - | - |
| 39 | - | **51** | - | - |
| 40 | - | 43 | - | - |
| 41 | - | **16** | - | - |
| 42 | - | **51** | - | - |
| 43 | - | **52** | - | - |
| 44 | - | 90 | - | - |
| 45 | - | 114 | - | - |
| 46 | - | **52** | - | - |
| 47 | - | 81 | - | - |
| 48 | - | **39** | - | - |
| 49 | - | **68** | - | - |
| 50 | - | **52** | - | - |
| 51 | - | **59** | - | - |
| 52 | - | 81 | - | - |
| 53 | - | 30 | - | - |
| 54 | - | **18** | - | - |
| 55 | - | - | **68** | - |
| 56 | - | - | **18** | - |
| 57 | - | - | 90 | - |
| 58 | - | - | 43 | - |
| 59 | - | - | 53 | - |
| 60 | - | - | **52** | - |
| 61 | - | - | - | **18** |
| 62 | - | - | - | **18** |
| 63 | - | - | - | **56** |
| 64 | - | - | - | **18** |
| 65 | - | - | - | 53 |
| 66 | - | - | - | 42 |
| 67 | - | - | - | 66 |
| 68 | - | - | - | 11 |
| 69 | - | - | - | **18** |

Bold number: high-risk HPV genotype

Underline number: possibly high-risk HPV genotype

**Additional file 1: Table S2.** Risk factors for overall HPV infection

| **Factors** | **Infections/cases** |  | **Unadjusted OR**  **(95% CI)** | **P** |  | **Adjusted OR**  **(95% CI)** | **P** |
| --- | --- | --- | --- | --- | --- | --- | --- |
| Age |  |  |  |  |  |  |  |
| Older age | 69/198 |  | 1.04 (1.02–1.07) | **< 0.01** |  | 1.04 (1.02-1.07) | **< 0.01** |
| Marital status |  |  |  |  |  |  |  |
| No | 24/75 |  | 1 |  |  |  |  |
| Yes | 45/123 |  | 1.23 (0.67–2.25) | 0.51 |  | – | – |
| Occupation |  |  |  |  |  |  |  |
| Unstable or no job | 16/53 |  | 1 |  |  |  |  |
| Blue collar | 14/47 |  | 0.98 (0.42–2.31) | 0.97 |  | – | – |
| White collar | 39/98 |  | 1.53 (0.75–3.12) | 0.24 |  | – | – |
| Education |  |  |  |  |  |  |  |
| < College | 22/84 |  | 1 |  |  | 1 |  |
| ≥ College | 47/114 |  | 1.98 (1.07–3.65) | **0.03** |  | 2.02 (1.06-3.84) | **0.03** |
| Smoking |  |  |  |  |  |  |  |
| No | 43/123 |  | 1 |  |  |  |  |
| Yes | 26/75 |  | 0.99 (0.54–1.80) | 0.97 |  | – | – |
| Alcohol consumption |  |  |  |  |  |  |  |
| No | 6/24 |  | 1 |  |  |  |  |
| Yes | 63/174 |  | 1.70 (0.64–4.51) | 0.28 |  | – | – |
| Family member with cancer |  |  |  |  |  |  |  |
| No | 62/175 |  | 1 |  |  |  |  |
| Yes | 7/23 |  | 0.80 (0.31–2.04) | 0.64 |  | – | – |
| STI knowledge |  |  |  |  |  |  |  |
| Yes | 64/188 |  | 1 |  |  | 1 |  |
| No | 5/10 |  | 1.94 (0.54-6.94) | 0.31 |  | 2.88 (0.76-10.87) | 0.12 |
| Circumcision |  |  |  |  |  |  |  |
| No | 48/139 |  | 1 |  |  |  |  |
| Yes | 21/59 |  | 1.05 (0.55–1.98) | 0.89 |  | – | – |
| Age at sexual debut |  |  |  |  |  |  |  |
| Older age | 69/198 |  | 1.03 (0.94–1.13) | 0.48 |  | – | – |
| Total number of sexual partners |  |  |  |  |  |  |  |
| Higher number | 69/198 |  | 1.00 (0.99–1.02) | 0.75 |  | – | – |
| Number of sexual partners  in last 6 months |  |  |  |  |  |  |  |
| Higher number | 169/98 |  | 0.97 (0.90–1.05) | 0.48 |  | – | – |
| Female sex worker as a sexual  partner in last 6 months |  |  |  |  |  |  |  |
| No | 57/164 |  | 1 |  |  |  |  |
| Yes | 12/34 |  | 1.02 (0.47–2.22) | 0.95 |  | – | – |
| Condom usage |  |  |  |  |  |  |  |
| Rarely or Never | 13/48 |  | 1 |  |  |  |  |
| Sometimes | 41/104 |  | 1.75 (0.83–3.70) | 0.14 |  | – | – |
| Everytime | 15/46 |  | 1.30 (0.54–3.16) | 0.56 |  | – | – |
| STI history |  |  |  |  |  |  |  |
| No | 19/66 |  | 1 |  |  |  |  |
| Yes | 50/132 |  | 1.51 (0.80–2.86) | 0.21 |  | – | – |
| Urethritis |  |  |  |  |  |  |  |
| No | 8/27 |  | 1 |  |  |  |  |
| Yes | 61/171 |  | 1.32 (0.54–3.19) | 0.54 |  | – | – |
| Oral sex |  |  |  |  |  |  |  |
| No | 46/126 |  | 1 |  |  |  |  |
| Yes | 23/72 |  | 0.82 (0.44–1.51) | 0.52 |  | – | – |

STI: sexually transmitted infection; OR: odds ratio; CI: confidence interval.

Dash (─) indicates not included in the last step of the stepwise binary logistic regression analysis
